# Correspondence

**Published:** 1881-08

**Authors:** 


### CORRESPONDENCE.

Chicago, July 6, 1881.

At a meeting of the Chicago Dental Society, on the 6th ult., the following was unanimously adopted:

M. S. Dean, Cor. Sec'y.

By F. F. F.

The Chicago Dental Society having learned of the recent death of Prof. James Taylor, of Cincinnati, desires to express its sense of the great loss which the profession has thereby sustained.

Dr. Taylor was among the foremost men of his day—one of the founders of the Ohio Dental College (the second institution of this kind organized in the world), and was ever found in the front ranks of those desiring the advancement of the profession.

Many members of this Society have enjoyed his personal friendship, and will always remember with feelings of pleasure his genial and kindly manners. His loss will be sincerely mourned, and he will long be remembered for his great usefulness and high moral and professional character.

*Resolved*, That the Secretary be instructed to forward to the Faculty of the Ohio Dental College, the DENTAL REGISTER, and the family of the deceased, a copy of this brief tribute to his memory.

